# Personalised progression prediction in patients with monoclonal gammopathy of undetermined significance or smouldering multiple myeloma (PANGEA): a retrospective, multicohort study

**DOI:** 10.1016/S2352-3026(22)00386-6

**Published:** 2023-02-27

**Authors:** Annie Cowan, Federico Ferrari, Samuel S Freeman, Robert Redd, Habib El-Khoury, Jacqueline Perry, Vidhi Patel, Priya Kaur, Hadley Barr, David J Lee, Elizabeth Lightbody, Katelyn Downey, David Argyelan, Foteini Theodorakakou, Despina Fotiou, Christine Ivy Liacos, Nikolaos Kanellias, Selina J Chavda, Louise Ainley, Viera Sandecká, Lenka Pospíšilová, Jiri Minarik, Alexandra Jungova, Jakub Radocha, Ivan Spicka, Omar Nadeem, Kwee Yong, Roman Hájek, Efstathios Kastritis, Catherine R Marinac, Meletios A Dimopoulos, Gad Get, Lorenzo Trippa, Irene M Ghobrial

**Affiliations:** a Medical Oncology, Dana-Farber Cancer Institute, Boston, MA, USA; b Bioinformatics Program, Broad Institute of MIT and Harvard, Cambridge, MA, USA; c Biostatistics and Research Decision Sciences, Merck & Co, Rahway, NJ, USA; d Department of Medicine, Massachusetts General Hospital, Boston, MA, USA; e Department of Clinical Therapeutics, National and Kapodistrian University of Athens, Athens, Greece; f UCL Cancer Institute, University College London, London, UK; g Department of Internal Medicine, Hematology and Oncology, University Hospital Brno, Brno, Czech Republic; h Institute of Biostatistics and Analyses, Brno, Czech Republic; i Department of Hemato-Oncology, University Hospital Olomouc, Olomouc, Czech Republic; j Department of Hematology and Oncology, University Hospital Pilsen, Pilsen, Czech Republic; k Fourth Department of Internal Medicine Hematology, Faculty of Medicine in Hradec Kralove, University Hospital Hradec Kralove, Charles University, Czech Republic; l First Department of Medicine, Department of Hematology, First Faculty of Medicine, Charles University and General Hospital in Prague, Czech Republic; m Fourth Department of Internal Medicine-Hematology, University Hospital in Ostrava, Ostrava, Czech Republic

## Abstract

**Background:**

Patients with precursors to multiple myeloma are dichotomised as having monoclonal gammopathy of undetermined significance or smouldering multiple myeloma on the basis of monoclonal protein concentrations or bone marrow plasma cell percentage. Current risk stratifications use laboratory measurements at diagnosis and do not incorporate time-varying biomarkers. Our goal was to develop a monoclonal gammopathy of undetermined significance and smouldering multiple myeloma stratification algorithm that utilised accessible, time-varying biomarkers to model risk of progression to multiple myeloma.

**Methods:**

In this retrospective, multicohort study, we included patients who were 18 years or older with monoclonal gammopathy of undetermined significance or smouldering multiple myeloma. We evaluated several modelling approaches for predicting disease progression to multiple myeloma using a training cohort (with patients at Dana-Farber Cancer Institute, Boston, MA, USA; annotated from Nov, 13, 2019, to April, 13, 2022). We created the PANGEA models, which used data on biomarkers (monoclonal protein concentration, free light chain ratio, age, creatinine concentration, and bone marrow plasma cell percentage) and haemoglobin trajectories from medical records to predict progression from precursor disease to multiple myeloma. The models were validated in two independent validation cohorts from National and Kapodistrian University of Athens (Athens, Greece; from Jan 26, 2020, to Feb 7, 2022; validation cohort 1), University College London (London, UK; from June 9, 2020, to April 10, 2022; validation cohort 1), and Registry of Monoclonal Gammopathies (Czech Republic, Czech Republic; Jan 5, 2004, to March 10, 2022; validation cohort 2). We compared the PANGEA models (with bone marrow [BM] data and without bone marrow [no BM] data) to current criteria (International Myeloma Working Group [IMWG] monoclonal gammopathy of undetermined significance and 20/2/20 smouldering multiple myeloma risk criteria).

**Findings:**

We included 6441 patients, 4931 (77%) with monoclonal gammopathy of undetermined significance and 1510 (23%) with smouldering multiple myeloma. 3430 (53%) of 6441 participants were female. The PANGEA model (BM) improved prediction of progression from smouldering multiple myeloma to multiple myeloma compared with the 20/2/20 model, with a C-statistic increase from 0·533 (0·480–0·709) to 0·756 (0·629–0·785) at patient visit 1 to the clinic, 0·613 (0·504–0·704) to 0·720 (0·592–0·775) at visit 2, and 0·637 (0·386–0·841) to 0·756 (0·547–0·830) at visit three in validation cohort 1. The PANGEA model (no BM) improved prediction of smouldering multiple myeloma progression to multiple myeloma compared with the 20/2/20 model with a C-statistic increase from 0·534 (0·501–0·672) to 0·692 (0·614–0·736) at visit 1, 0·573 (0·518–0·647) to 0·693 (0·605–0·734) at visit 2, and 0·560 (0·497–0·645) to 0·692 (0·570–0·708) at visit 3 in validation cohort 1. The PANGEA models improved prediction of monoclonal gammopathy of undetermined significance progression to multiple myeloma compared with the IMWG rolling model at visit 1 in validation cohort 2, with C-statistics increases from 0·640 (0·518–0·718) to 0·729 (0·643–0·941) for the PANGEA model (BM) and 0·670 (0·523–0·729) to 0·879 (0·586–0·938) for the PANGEA model (no BM).

**Interpretation:**

Use of the PANGEA models in clinical practice will allow patients with precursor disease to receive more accurate measures of their risk of progression to multiple myeloma, thus prompting for more appropriate treatment strategies.

**Funding:**

SU2C Dream Team and Cancer Research UK.

**Research in context**

**Evidence before this study**

Prediction models are used to predict future outcomes through the analysis of large datasets. We searched for evidence of time-varying prediction models in precursor disease through PubMed, Google Scholar, and MEDLINE from database inception to April 31, 2022, in the English language. Terms included in this search were “monoclonal gammopathy of undetermined significance”, “MGUS”, “smoldering multiple myeloma”, “SMM”, “multiple myeloma”, “progression”, “prediction”, and “modeling”. Results included primarily analyses of current standard risk criteria for precursor disease progression. There were no prediction models that used multivariable, time-varying biomarkers to predict the risk of precursor disease progression to multiple myeloma.

**Added value of this study**

The PANGEA project is, to our knowledge, the largest international project of time-varying biomarker data on patients with precursors to multiple myeloma. Our findings show that the PANGEA models are more accurate than current precursor progression risk criteria including the International Myeloma Working Group (IMWG) risk stratification for monoclonal gammopathy of undetermined significance and the 20/2/20 risk stratification for smouldering multiple myeloma. These accuracy improvements were also demonstrated in large, independent validation cohorts.

**Implications of all the available evidence**

The improved accuracy of the PANGEA models over current risk criteria suggests that models that incorporate dynamic measurements of myeloma-specific parameters can improve clinician's ability to make therapeutic decisions for individual patients. The PANGEA models can be directly accessed in clinic and are appropriate replacements of the IMWG risk stratification criteria for patients with monoclonal gammopathy of undetermined significance and 20/2/20 risk criteria for patients with smouldering multiple myeloma.

## Introduction

Multiple myeloma is often preceded by two precursor conditions, monoclonal gammopathy of undetermined significance and smouldering multiple myeloma, with current diagnostic criteria differentiating these from symptomatic multiple myeloma,^1^, ^2^, ^3^, ^4^ as defined by SLiM-CRAB guidelines: clonal bone marrow plasma cells greater than or equal to 60%; serum free light chain (FLC) ratio greater than or equal to 100, provided involved FLC level is 100 mg/L or higher; more than one focal lesion on MRI; hypercalcaemia; renal failure; anaemia; and bone lesions.^5^ Various criteria have been developed to stratify patients with precursor disease into risk groups based on predicted probability of progression to multiple myeloma and to identify which patients might benefit from early intervention. The Mayo criteria stratify patients with smouldering multiple myeloma into risk categories depending on no risk factors (low-risk), one risk factor (intermediate-risk), or two or more risk factors (high-risk), which include a free light chain (FLC) ratio of more than 20, a monoclonal protein concentration of more than 2·0 g/dL, and a bone marrow plasma cell percentage (BMPC%) of more than 20%.^6^ This 20/2/20 stratification system was updated by the International Myeloma Working Group (IMWG) to include the fluorescence in-situ hybridisation (FISH) results of t(4;14), t(14;16), gain(1q), and del(13/13q).^7^ These models are applied at precursor diagnosis and rely on discrete cutoffs despite inherent variation in biomarkers throughout disease monitoring.^8^, ^9^ Consequently, the models are rarely used to restratify patients according to evolving laboratory findings,^8^, ^9^ despite improvements to the ability of the 20/2/20 model to prognosticate when applied at discrete timepoints after diagnosis.^10^

Current risk stratification criteria are also limited by variation in the availability and measurement of bone marrow biomarkers. Smouldering multiple myeloma progression risk is often estimated using BMPC%, and the arbitrary cutoff of 10% BMPC is used to dichotomise monoclonal gammopathy of undetermined significance and smouldering multiple myeloma. However, the use of discrete BMPC% categories is limited by heterogeneity of the involved marrow, an absence of early-stage biopsies, and heterogeneous interpretations by pathologists.^11^, ^12^ Previous studies have shown that some rates of change of biomarkers more accurately predict progression than a discrete value at a single timepoint. For example, evolving M-protein (monoclonal protein) and haemoglobin concentrations were independent predictors of progression within 2 years for patients with smouldering multiple myeloma.^13^ Also, Markov models of longitudinal data enhance predictions of myeloproliferative disease progression.^14^ These studies suggest a need for the development and validation of prediction models that incorporate time-varying biomarkers to update risk throughout precursor evolution and to prognosticate time to progression, particularly for haematological diseases that rely heavily on longitudinal serum measurements.

To address this need, we developed the Precursor Asymptomatic Neoplasms by Group Effort Analysis (PANGEA) model*,* which uses time-varying clinical biomarkers to model how precursor progression risk to multiple myeloma evolves for a single patient over time, both with and without bone marrow biopsies. We assembled a cohort of patients with monoclonal gammopathy of undetermined significance and patients with smouldering multiple myeloma with serial laboratory measurements and we developed multivariate Cox models with time-varying patient profiles to predict precursor progression to multiple myeloma. Our hypothesis is that disease progression from monoclonal gammopathy of undetermined significance or smouldering multiple myeloma to overt multiple myeloma can be anticipated by trends in clinical values that are associated with clonal proliferation and that modelling these changes can improve predictions of progression risk. We strove to develop models with commonly available biomarkers to allow for broad clinical application, and we validated these models in two independent cohorts. This validation illustrates that both PANGEA models (with [BM] and without bone marrow biopsy [no BM]) outperform the prediction accuracy of previous models in multiple cohorts. Finally, we provide an online calculator implementing the PANGEA model that allows clinicians and patients to assess individual risk of progression and consider early therapeutic interceptions.

## Methods

### Study design

In this retrospective, multicohort study, we included an international cohort of patients with precursor disease to multiple myeloma with serial clinical and biological variables. Patients were identified retrospectively at oncology centres (Dana-Farber Cancer Institute [DFCI; Boston, MA, USA], National and Kapodistrian University of Athens [Athens, Greece], University College London [UCL; London, UK]), and the cancer group Registry of Monoclonal Gammopathies (RMG; Czech Republic).

This study was approved by the DFCI Institutional Review Board (21–127) and done in accordance with the Declaration of Helsinki. Consent was waived due to the non-invasive nature of this research.

### Participants

The PANGEA project included patients with smouldering multiple myeloma and monoclonal gammopathy of undetermined significance within three independent cohorts: the training cohort, which included patients at DFCI (annotated from Nov 13, 2019, to April 13, 2022); the validation cohort 1, which included patients at University of Athens (annotated from Jan 26, 2020, to Feb 7, 2022) and patients at UCL (annotated from May 9, 2020, to April 10, 2022); and validation cohort 2, which included patients at RMG (annotated from May 1, 2004, to March 10, 2022. For more information on the cohorts see appendix (p 1).

Patients from all four sites were eligible for inclusion if aged 18 years or older, diagnosed with non-IgM monoclonal gammopathy of undetermined significance or smouldering multiple myeloma by the IMWG criteria. Patients diagnosed with overt multiple myeloma at diagnosis were excluded from analysis, and patients treated with therapy during their precursor disease course were censored at treatment start dates. Patients were included in analysis until the date of progression per SLiM-CRAB criteria, death, or initiation of treatment. In all three cohorts, patients were selected for analysis from tissue-banking and retrospective monitoring trials for precursor disease states.

### Procedures

The time of diagnosis and the first visit (visit 1) coincided in all cohorts (ie, the average time between date of original diagnosis and visit 1 was 0 months for training cohort, validation cohort 1, and validation cohort 2).

We retrieved patient information for total protein, IgA via nephelometry, IgM, IgG, κ-free light chain (FLC) and λ-FLC via Optilite (Binding Site, Birmingham, UK), FLC ratio (involved and uninvolved), calcium, creatinine, albumin, haemoglobin, lactate dehydrogenase, β2-microglobulin, M-protein, and bodyweight from medical records. Serial values were annotated on average at 5 (IQR 3–8) month time intervals from the date of monoclonal gammopathy of undetermined significance or smouldering multiple myeloma diagnosis, censoring at the date of progression to active multiple myeloma, last follow-up, initiation of precursor treatment, or death. We also retrieved data on gender, race, ethnicity, age at diagnosis, height, progression, survival status, immunofixation isotype, and bisphosphonate use. For all bone marrow biopsies, plasma cell percentages were collected from core biopsy samples and FISH results from bone marrow aspirates (appendix p 4).

We built the PANGEA model, a multivariate Cox regression with time-varying biomarkers, by selecting clinically significant predictors of progression (age, FLC ratio, M spike in g/dL, creatinine in mg/dL, and BMPC%) identified using the training cohort. FLC ratio and creatinine concentration were log-transformed to reduce outlier effect. We also evaluated whether biomarker trends correlated with the progression risk and selected decreasing haemoglobin concentration as a categorical trend variable (appendix p 3). We compared the predictive accuracy of this model with those created through backward selection and Bayes information criterion and selected the most accurate model containing the least redundancy.

We developed two versions of the PANGEA model (BM and no BM). Our final Cox model (named the PANGEA model [BM]) included age, FLC ratio, M spike concentration in g/dL, creatinine concentration in mg/dL, BMPC%, and the haemoglobin trajectory variable (appendix p 14). We then eliminated all biomarkers that require a bone marrow biopsy and repeated the modelling process (the PANGEA model [no BM]) with four continuous predictors (age, FLC ratio, M spike concentration in g/dL, and creatinine concentration in mg/dL, and haemoglobin trajectory; appendix p 14). The models assume that the hazard of progression to multiple myeloma is a linear function that only depends on a patient's clinical profile and is conditional on expected time to death.

We developed a web application that allows input of patient variables of the PANGEA model (BM and no BM) using the Shiny R package (1.7.1). The resulting PANGEA app outputs a patient's risk of progression using these biomarkers (monoclonal protein, involved over uninvolved FLC ratio, creatinine, haemoglobin trajectory, and age; appendix p 5). Alternatively, if bone marrow data is not available, users can enter all other variables, and patient progression risk will be evaluated using the PANGEA (no BM) model. If longitudinal measurements are available, users can enter variables at multiple time points.

The main outcome measure, time to progression, was defined as the time from precursor disease diagnosis per IMWG criteria^4^ to multiple myeloma diagnosis per SLiM-CRAB^5^ criteria.

### Statistical analysis

We used bootstrapping and calibration analyses (appendix pp 16, 21) and Schoenfeld tests, residual plots, and splines of predictors (appendix pp 11, 19–20) to assess the PANGEA models. R (version 4.2.0) was used for all statistical analyses. The average number of timepoints for validation cohort 1 was six and for validation cohort 2 was one; thus, we used validation cohort 1 to validate how the PANGEA model performed for patients with follow-up and validation cohort 2 to validate how the PANGEA model performed at diagnosis (visit 1). When comparing the PANGEA model with the current risk stratification criteria, application of the IMWG^4^ or 20/2/20^6^ criteria as binary cutoffs at diagnosis will be referred to as the baseline model and restratification by these criteria as discrete variables over time will be referred to as the rolling model. Subcohorts of patients with smouldering multiple myeloma from validation cohort 1 and validation cohort 2 were used for comparative analyses against the baseline and rolling 20/2/20 models. A subcohort of patients with monoclonal gammopathy of undetermined significance from validation cohort 2 was used for comparative analyses against the baseline and rolling IMWG models.

The C-statistic is a standard metric used to compare prediction models. A C-statistic of 0·5 indicates that the model performs no better than random chance and a C-statistic of 1 indicates perfect prediction. For the PANGEA models, we computed C-statistics for visits 1, 2, and 3 for validation cohort 1 and at visit 1 for validation cohort 2. For the baseline models, we fit a Cox model in the training cohort to estimate the hazard ratios (HRs) for risk groups and computed the Cox linear combination of predictor and C-statistics in the validation cohorts. For the rolling models, we fit a time-varying Cox model in the training cohort to estimate HRs and computed the C-statistics at visits 1, 2, and 3 in validation cohort 1. The C-statistic estimates for validation cohort 1 and validation cohort 2 are representative of model accuracy in two cohorts independent from the training cohort used for developing the PANGEA models.

To visualise the time to progression for the validation cohorts, we divided patients into quartiles (low, intermediate-low, intermediate-high, and high risk) based on their predicted risk from the PANGEA models. This discretisation is only used when needed for graphical summaries and for comparisons with models that define risk groups. We visualised these groups using Kaplan-Meier curves for time to progression or death (with patients censored at treatment). In these analyses, we included patients who qualified for the PANGEA models by having all necessary biomarker values available at the visit of interest.

We explored whether FISH biomarkers could provide additional prediction improvements to the PANGEA model (BM). Due to the frequent absence or failure of FISH testing and the rarity of some cytogenetic alterations, our training cohort was of small size. Therefore, we selected patients with one or more successful FISH panels and corresponding laboratory datasets, resulting in a subcohort of patients (appendix pp 8–9). We built the PANGEA model (FISH) by selecting significant predictors.

### Role of the funding source

The funder of the study had no role in study design, data collection, data analysis, data interpretation, or writing of the report.

## Results

**Figure 1:**
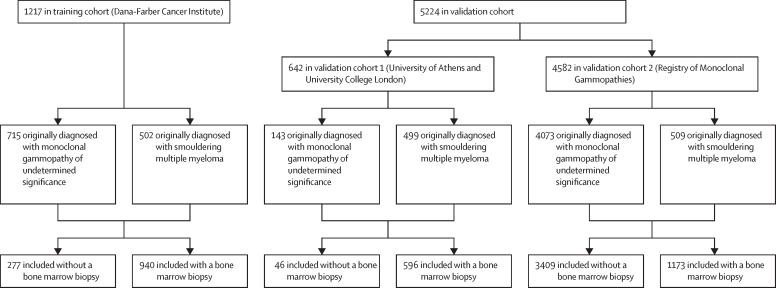
Patient flow in the training and validation cohorts of the PANGEA project

The training cohort comprised 1217 patients (715 with monoclonal gammopathy of undetermined significance and 502 with smouldering multiple myeloma, with 172 progressing to multiple myeloma); validation cohort 1 comprised 533 patients (143 with monoclonal gammopathy of undetermined significance and 390 with smouldering multiple myeloma, with 112 progressing to multiple myeloma) at University of Athens and 109 patients with smouldering multiple myeloma (with 31 progressing to multiple myeloma) at UCL; and validation cohort 2 comprised 4582 (4073 monoclonal gammopathy of undetermined significance and 509 smouldering multiple myeloma, with 745 progressing to multiple myeloma) at RMG (table 1, figure 1). The distribution of biomarkers within the training cohort is summarised across 20/2/20 risk groups (appendix p 7). The median number of timepoints (clinic visits) was seven (range one to 40) for the training cohort, six (range one to 40) for the validation cohort 1, and one (range one to one) for validation cohort 2. The median follow-up time was 4·2 (IQR 0·0–30·5) years for the training cohort, 2·9 (IQR 0·0–21·4) years for the validation cohort 1, and 3·6 (IQR 0·0–73·9) years for validation cohort 2. Validation cohort 1 had a similar progression proportion (2·23% [95% CI 1·19–3·79]), defined as the proportion of patients who progressed to multiple myeloma within three clinical visits, to the training cohort (2·18% [1·35–3·10]), whereas validation cohort 2 had a lower proportion of those who had disease progression (0·11% [0·03–0·28]).

**Table 1:** Patient demographics of training and validation cohorts of the PANGEA project

		**Total (n=6441)**	**Training cohort (n=1217)**	**Validation cohort 1 (n=642)**	**Validation cohort 2 (n=4582)**
Age at initial diagnosis, years		64·22 (19·52– 94·00)	62·00 (22·00–94·00)	64·00 (28·50–89·09)	65·21 (19·52–93·77)
	Missing	42 (1%)	0	0	42 (1%)
Clinical laboratory visits		7 (1–40)	7 (1–40)	6 (1–40)	1 (1–1)
Interval between visits, months		5 (0–140)	6 (0–140)	5 (0–103)	5 (0–112)
Sex					
	Female	3430 (53%)	642 (53%)	374 (58%)	2414 (53%)
	Male	3009 (47%)	575 (47%)	266 (41%)	2168 (47%)
	Missing	2 (0%)	0	2 (0%)	0
Race					
	White	1575 (24%)	992 (82%)	583 (91%)	0
	Black or African American	156 (2%)	137 (11%)	19 (3%)	0
	Asian	45 (1%)	28 (2%)	17 (3%)	0
	Multiple	7 (0%)	6 (0%)	1 (0%)	0
	Declined	9 (0%)	8 (1%)	1 (0%)	0
	Other	37 (1%)	26 (2%)	11 (2%)	0
	Missing	4612 (72%)	20 (2%)	10 (2%)	4582 (100%)
Ethnicity					
	Declined	8 (0%)	8 (1%)	0	0
	Not Hispanic or Latino	1053 (16%)	1052 (86%)	1 (0%)	0
	Hispanic or Latino	54 (1%)	54 (4%)	0	0
	Missing	5326 (83%)	103 (8%)	641 (100%)	4582 (100%)
Original diagnosis					
	Monoclonal gammopathy of undetermined significance	4931 (77%)	715 (59%)	143 (22%)	4073 (89%)
	Smouldering multiple myeloma	1510 (23%)	502 (41%)	499 (78%)	509 (11%)
Progression to smouldering multiple myeloma					
	Not progressed to smoldering multiple myeloma	4520 (70%)	437 (36%)	138 (21%)	3945 (86%)
	Progressed to smouldering multiple myeloma	411 (6%)	278 (23%)	5 (1%)	128 (3%)
	Smouldering multiple myeloma as original diagnosis	1510 (23%)	502 (41%)	499 (78%)	509 (11%)
Progression to multiple myeloma					
	Not progressed to multiple myeloma	5381 (84%)	1045 (86%)	499 (78%)	3837 (84%)
	Progressed to multiple myeloma	1060 (16%)	172 (14%)	143 (22%)	745 (16%)
Immunofixation					
	IgG	4908 (76%)	882 (72%)	462 (72%)	3564 (78%)
	IgA	1127 (17%)	232 (19%)	149 (23%)	746 (16%)
	Light chain only	179 (3%)	75 (6%)	14 (2%)	90 (2%)
	Biclonal	34 (1%)	21 (2%)	9 (1%)	4 (0%)
	Missing	193 (3%)	7 (1%)	8 (1%)	178 (4%)
Died					
	No	5080 (79%)	1133 (93%)	569 (89%)	3405 (74%)
	Yes	1334 (21%)	84 (7%)	73 (11%)	1177 (26%)
Censored for treatment					
	Yes	229 (4%)	222 (18%)	6 (1%)	1 (0%)
	No	1370 (21%)	995 (82%)	636 (99%)	4581 (0%)

Data are in n (%) or median (range).

Variations in accuracy between the three modelling processes (significant predictor selection, backward selection, and Bayes information criterion), as measured by C-statistics, were less than 2%. All variables selected for these models were identical except for the PANGEA model (no BM) produced by Bayes information criterion, which incorporated albumin and isotype. We selected the significant predictor model due to its accuracy and succinctness.

**Figure 2:**
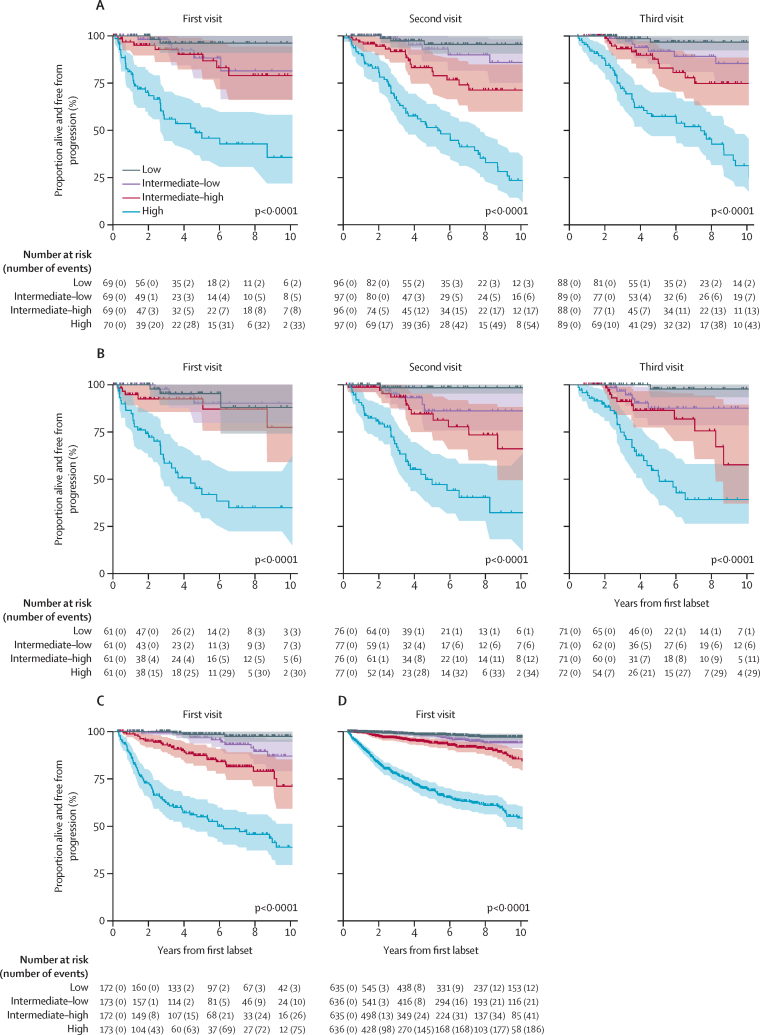
Time to progression predictions from precursor disease In validation cohort 1 using (A) PANGEA (no BM) model (visit 1, n=70; visit 2, n=97; visit 3, n=89) and (B) PANGEA (BM) model (visit 1, n=61; visit 2, n=77; visit 3, n=72). In validation cohort 2 using (C) PANGEA (BM) model (n=173) and (D) PANGEA (no BM) model (n=636) at visit 1. PANGEA (BM)=PANGEA model with bone marrow biopsy. PANGEA (no BM)=PANGEA model without bone marrow biopsy RMG=Registry of Monoclonal Gammopathies.

FLC ratio, M-spike concentration, age, creatinine concentration, BMPC%, and haemoglobin trajectory were used in the PANGEA model (BM; figure 2; appendix p 14). Decreases in haemoglobin levels were significantly associated with increased risk. Although there was an expected average difference in baseline haemoglobin concentrations between male (mean 14·4 [SD 14·5 to 14·6] g/dL) and female (12·9 [12·8 to 13·0] g/dL; p<0·0001 for patients who did not progress to multiple myeloma) patients, there was no significant difference in the rate of change in haemoglobin concentration between male (point estimate –0·24 [SD –0·22 to –0·26] g/dL per year) and female patients (–0·24 [–0·22 to –0·26] g/dL per year; p=0·83 for the training cohort. Similar results were observed in validation cohort 1 and validation cohort 2 (appendix p 15).

The PANGEA model (no BM) included haemoglobin trajectory, FLC ratio, M spike concentration, age, and creatinine concentration as significant progression predictors (appendix p 14). Total protein, κ-FLC or λ-FLC, calcium (corrected for albumin) concentration, LDH, and β2-microglobulin concentrations, and bisphosphonate use, family history of haematological malignancy, time with disease, race, ethnicity, and sex were not significant indicators of disease progression.

The PANGEA model improved prediction of smouldering multiple myeloma progression to multiple myeloma compared with both 20/2/20 models (baseline and rolling) in validation cohort 1 and validation cohort 2, as indicated by a C-statistic increase of more than 10% (table 2). The PANGEA (BM) model had an increase in C-statistic from the baseline model of 42% (from 0·533 [95% CIs 0·480–0·709] to 0·756 [0·629–0·785]) and an increase of 18% from the rolling model (from 0·613 [0·504–0·704] to 0·720 [0·592–0·775] at visit two and from 0·637 [0·386–0·841] to 0·756 [0·547–0·830] at visit three) in validation cohort 1 (table 2). Similarly, the PANGEA (no BM) model showed a 30% increase (from 0·534 [0·501–0·672] to 0·692 [0·614–0·736]) in C-statistic compared with the baseline model and an average increase of 22% (from 0·573 [0·518–0·647] to 0·693 [0·605–0·734] at visit two and from 0·560 [0·497–0·645] to 0·692 [0·570–0·708] at visit three) compared with the rolling model in validation cohort 1 (table 2). For validation cohort 2, there was a 22% (from 0·502 [0·482–0·604] to 0·610 [0·525–0·931]) increase in the PANGEA model (BM) and 45% (from 0·492 [0·460–0·561] to 0·714 [0·589–0·933]) increase in the PANGEA model (no BM) in C-statistic compared with the baseline model (table 2).

**Table 2:** Performance of the PANGEA models compared with the baseline and rolling 20/2/20 models in patients with smouldering multiple myeloma

	**Baseline model (20/2/20)**		**Rolling model (20/2/20)**		**PANGEA models**	
	No bone marrow	Bone marrow	No bone marrow	Bone marrow	No bone marrow	Bone marrow
Validation cohort 1, visit 1	0·534 (0·501–0·672)	0·533 (0·480–0·709)	0·625 (0·526–0·649)	0·669 (0·537–0·696)	0·692 (0·614–0·736)	0·756 (0·629–0·785)
Validation cohort 1, visit 2	..	..	0·573 (0·518–0·647)	0·613 (0·504–0·704)	0·693 (0·605–0·734)	0·720 (0·592–0·775)
Validation cohort 1, visit 3	..	..	0·560 (0·497–0·645)	0·637 (0·386–0·841)	0·692 (0·570–0·708)	0·756 (0·547–0·830)
Validation cohort 2 visit 1	0·492 (0·460–0·561)	0·502 (0·482–0·604)	0·492 (0·472–0·536)	0·502 (0·472–0·568)	0·714 (0·589–0·933)	0·610 (0·525–0·931)

Data shown are C-statistic (95% CI), as tested in patients with smouldering multiple myeloma from validation cohort 1 and validation cohort 2. Bootstrapping is shown on appendix (p 16).

The PANGEA models outperformed the rolling IMWG model for patients with monoclonal gammopathy of undetermined significance with improvements of 24% (from 0·640 [0·518–0·718] to 0·729 [0·643–0·941]), C-statistics from the PANGEA model (BM), and 31% (from 0·670 [0·523–0·729] to 0·879 [0·586–0·938]) from the PANGEA model (no BM) in validation cohort 2 (appendix p 10).

**Figure 3:**
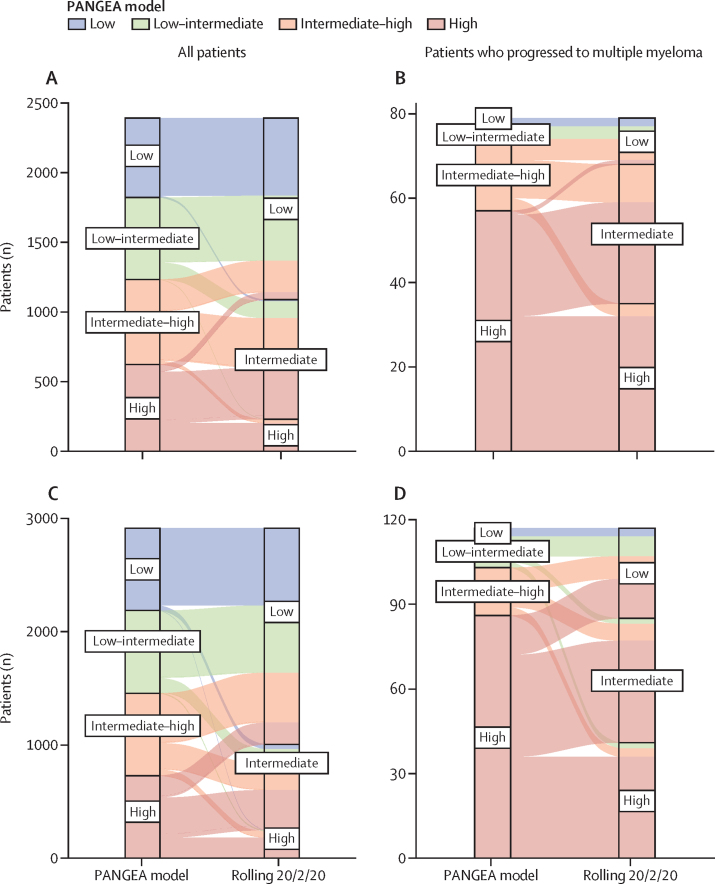
Risk stratification of the PANGEA models compared with the rolling 20/2/20 model in the validation cohorts at visit 1 (A) All patients using the PANGEA model (BM). (B) All patients who had progression to multiple myeloma using the PANGEA model (BM). (C) All patients using the PANGEA (no BM) model. (D) Patients who had progression to multiple myeloma using the PANGEA (no BM) model. PANGEA (BM)=PANGEA model with bone marrow biopsy. PANGEA (no BM)=PANGEA model without bone marrow biopsy. Rolling model=20/2/20 criteria with restratification by these criteria as discrete variables over time.

The PANGEA models improved output probabilities of progression for individual patients with smouldering multiple myeloma in validation cohort 1 and validation cohort 2 and patients with monoclonal gammopathy of undetermined significance in validation cohort 2 (figure 3; appendix p 17) when they were artificially stratified into high, high-intermediate, low-intermediate, and low progression risk groups. We compared the predicted risk groups in validation cohort 1, and 58% of patients with smouldering multiple myeloma who eventually had progression to multiple myeloma were reclassified from a 20/2/20 intermediate-risk or low-risk category into a PANGEA (BM) high-risk category (figure 3B). Furthermore, patients who did not have progression to multiple myeloma were often classified with lower risks than those who do progress (figure 3A, 3C). Similarly, 43% of patients with monoclonal gammopathy of undetermined significance who eventually had progression to multiple myeloma were reclassified from a IMWG lower risk category into a PANGEA model (BM) high-risk category (appendix p 17).

Currently, bone marrow biopsies are the primary source of genomic information available from the clinic. Because genomic aberrations have a crucial role in precursor progression,^15^, ^16^ we expanded the PANGEA model (BM) to include FISH covariates. The resulting PANGEA model (FISH) used the significant predictors of age, FLC ratio, M spike concentration, creatinine concentration, BMPC%, del(17/17p), gain(1q), del(13/13q), and haemoglobin trajectory (appendix p 18). We also identified *MYC* rearrangement (8q24) as a significant covariate in a subcohort of 957 patients from the training cohort and validation cohort 1 who were tested for this translocation (appendix pp 8–9). The significance of FISH biomarkers suggests potential for further improvements to the PANGEA model when additional datasets for validation become available.

## Discussion

The study of precursor disease created stratification systems, which identify patients at the highest risk of progression to multiple myeloma. However, current monoclonal gammopathy of undetermined significance and smouldering multiple myeloma progression prediction algorithms stratify patients into risk groups using baseline measurements rather than time-varying biomarkers. Leading models, such as the 20/2/20 model^6^ and the PETHEMA criteria,^17^ do not align on which patients classify as at high risk.^18^ Discordant definitions of disease risk and an inability to update this risk over time have led to differences in clinical trial inclusion and treatment strategies for patients with precursor multiple myeloma. Large, new datasets of patients offer opportunities to evaluate progression risk with statistical models and to translate time-varying biomarkers into predictions that support clinical decisions.

We assembled a cohort of patients with precursor multiple myeloma with extensive longitudinal data to develop the PANGEA models, multivariate Cox regressions that use widely available, time-varying biomarkers with and without bone marrow data, to improve predictions of individual patients’ progression risk. The PANGEA models incorporate clinical variables beyond typical measures of tumour burden, including creatinine concentration, age, and haemoglobin concentration, in addition to those in the 20/2/20 criteria (M spike concentration, FLC ratio, and BMPC%). The parameters of the PANGEA models are concordant with recent research that found that decreasing haemoglobin is an independent predictor of smouldering multiple myeloma progression to multiple myeloma^19^ and decreased renal function at precursor diagnosis is associated with worse outcomes.^20^ Research has also shown that incidences of monoclonal gammopathy of undetermined significance, smouldering multiple myeloma, and multiple myeloma increase with age;^2^ the PANGEA models capture this distinction by incorporating an age variable. Additionally, dynamic assessment of risk was suggested by Blade and collegues^21^ as early as 1989 and, more recently, shown by the Mayo group with improvements to the 20/2/20 model's ability to prognosticate when reapplied after diagnosis.^10^ However, most of these studies have been small relative to the PANGEA project, have failed to include time-varying biomarkers, and have not been validated in external cohorts.^6^, ^7^

A crucial difference between PANGEA and the 20/2/20 risk criteria is that the PANGEA models provide patient-specific probabilities of progression. PANGEA allows for improved prognostication, as validation analyses showed a relative precision improvement over current risk criteria. When models are applied to the same cohort, C-statistics allowed for direct comparison of predictive accuracy. Analysis of the PANGEA model compared with the baseline and rolling 20/2/20 models for patients with smouldering multiple myeloma and the rolling IMWG for patients with monoclonal gammopathy of undetermined significance all showed changes in C-statistic of greater than 10%. This increase in C-statistic was validated by early identification of patients who later progressed to overt multiple myeloma, with 58% of progressors identified as high risk by the PANGEA model and not by the rolling 20/2/20 model (figure 3). Our comparisons to alternative stratification models highlight that the PANGEA models are clinically appropriate, improve prediction accuracy, and capture changes in disease risk after diagnosis.

A crucial goal of this project was to identify the role of bone marrow biopsies in risk prediction. Despite the reliance of current stratification models on BMPC%, many patients with precursors to multiple myeloma do not regularly undergo bone marrow biopsies or forgo them altogether. These patients cannot be adequately assessed by risk criteria that rely on BMPC%. The PANGEA model (no BM) shows that progression risk can be accurately estimated with trends in serum biomarkers. Specifically, both PANGEA models (BM and no BM) outperform the baseline and dynamic models for the IMWG monoclonal gammopathy of undetermined significance and 20/2/20 smouldering multiple myeloma criteria (appendix p 10, table 2). These data suggest that variables derived from bone marrow biopsies are not required to accurately determine progression risk. When bone marrow biopsy data are no longer required and with considerable biological overlap between monoclonal gammopathy of undetermined significance and smouldering multiple myeloma,^15^, ^16^, ^22^, ^23^ predictions models that consider these precursor conditions together are advantageous. With this approach, we foresee a transition from coarse, discrete risk groups (monoclonal gammopathy of undetermined significance *vs* smouldering multiple myeloma risk groups) to a granular spectrum of the precursor population at the individual level. Regardless of a patient's bone marrow status, the PANGEA model can be used via the online PANGEA app to easily calculate progression risk of all precursor patients.

Genomic and epigenetic factors that lead to multiple myeloma progression are also a crucial part of a patient's progression risk.^15^, ^16^, ^24^ Studies have shown that monoclonal gammopathy of undetermined significance and smouldering multiple myeloma clones already harbour chromosomal alterations and that progression to multiple myeloma is due to the expansion of clones that are present in early disease stages.^24^, ^25^, ^26^ We built the PANGEA model (FISH), which incorporated sequential cytogenetic data in personalised risk prediction. The PANGEA model (FISH) is novel in that it examines changes in cytogenetic alterations when providing probabilities of disease progression. The PANGEA model (FISH) model shows the predictive value of FISH variables and suggests that previously imperceptible clonal tumour evolution might be approximated by clinical cytogenetic results; however, future studies are required to evaluate this model in independent datasets.

Together, PANGEA is a three-tiered model (BM, no BM, and FISH), which can take advantage of complex clinical tests or be readily available for patients with few data. FISH and bone marrow biopsies were included in our analysis because we acknowledge that both physicians and patients will continue to request them; however, patients without bone marrow biopsies and FISH results can receive accurate risk predictions with the PANGEA model (no BM) as it also outperforms existing models*.*

The PANGEA models are inherently limited by the selected variables and modelling process, our prioritisation for model simplicity and interpretability, and our assumptions on proportional hazards and non-informative censoring. Larger datasets, advanced machine-learning, and extended validation cohorts have the potential to improve accuracy in the future. We plan to evaluate circulating tumour cells, cell-free DNA, immune variables, and other biomarkers to refine risk stratification. We also aim to use prospective cohorts for further validation and we look forward to ethically including more patients with precursors to multiple myeloma who identify as African American—a population with increased prevalence of precursor conditions. The hope is that the PANGEA models dramatically improve how clinicians can inform patients of their personalised risk of developing myeloma and aid decision making for early therapeutic interception, particularly when recommending follow-up testing to monitor time-varying biomarkers. The PANGEA model is freely accessible, using continuous variables available in all clinical settings, enabling its use at both the individual patient level and in clinical trials for the rapid development of therapeutic interventions.

## Data sharing

The PANGEA team encourages collaboration to further model development. Data from this project can be made available in aggregate and after deidentification to investigators who submit appropriate proposals approved by the study team. Please direct questions to irene_ghobrial@dfci.harvard.edu.

## Declaration of interests

This study was previously presented on April 12, 2022, at the 2022 American Association for Cancer Research Annual Meeting and on Aug 25, 2022, at the International Myeloma Society Annual Meeting. AC declares grants from the International Myeloma Society for travel and conference expenses. FF is employed by Biostatistics and Research Decision Sciences, Merck & Co. SSF declares that their salary is partly supported by research funding from International Business Machines (IBM) and has patent applications (EP14807512·0A and US16/084 890) and a provisional patent application (62/866 261). LA declares grants from the International Myeloma Society for travel and conference expenses. JR declares honoraria from Sanofi, Janssen, Amgen, GSK, and Bristol Myers Squibb; travel grants from BMS, Janssen, and Amgen; and funding from a consulting or advisory role from Sanofi, Janssen, Amgen, GSK, and BMS. EK reports honoraria from Amgen, Janssen, Takeda, Genesis Pharma, Pfizer, and GSK; travel grants from Janssen; and is an advisory board member at Janssen and Prothena. MAD declares honoraria from Amgen, BMS, Takeda, and Janssen and is an advisory board member at Amgen, BMS, Takeda, and Janssen. CRM reports research funding from GRAIL. GG declares honoraria for lectures from Society for Neuro-oncology, Society of Tumor Oncology, and MD Anderson; honoraria as a Paul C Zamecnik Chair in Oncology; research funding from IBM and Pharmacyclics; patents, royalties, other intellectual property as Inventor on patent applications related to MSMuTect, MSMutSig, MSIDetect, POLYSOLVER, and SignatureAnalyzer-GPU; and stock and other ownership interests from Founder as a consultant and has privately-held equity in Scorpion Therapeutics. IMG declares honoraria from Celgene, Bristol-Myers Squibb, Takeda, Amgen, Janssen, and Vor Biopharma; consulting or advisory roles at Bristol-Myers Squibb, Novartis, Amgen, Takeda, Celgene, Cellectar, Sanofi, Janssen, Pfizer, Menarini Silicon Biosystems, Oncopeptides, The Binding Site, GSK, AbbVie, Adaptive, and 10xGenomics; and a spouse who is the Chief Medical Officer at Disc Medicine and holds equity in the company. AC, FF, SSF, GG, LT, and IMG have applied for a patent for the application of the PANGEA models described in this paper.
