# Supplementary material for: Personalised progression prediction in patients with monoclonal gammopathy of undetermined significance or smouldering multiple myeloma (PANGEA): a retrospective, multicohort study
Source: Lancet Haematol. 2023 Feb 27;10(3):e203–12. doi: 10.1016/S2352-3026(22)00386-6 (PMC9991855; doi:10.1016/S2352-3026(22)00386-6)
Supplement: Supplementary appendix [file mmc1.pdf]

THE LANCET
Haematology

Supplementary appendix

This appendix formed part of the original submission and has been peer reviewed. We post it as supplied by the authors.

Supplement to: Cowan A, Ferrari F, Freeman SS, et al. Personalised progression prediction in patients with monoclonal gammopathy of undetermined significance or smouldering multiple myeloma (PANGEA): a retrospective, multicohort study. *Lancet Haematol* 2023; **10:** e203–12.

Appendix

The PANGEA Project

Patients from DFCI were identified by their inclusion in the PCROWD Study (NCT02269592) or by search in the Research Patient Data Registry (RPDR) of the Mass General Brigham (MGB) database for ICD9 codes c90.0 (SMM/MM) and d47.2 (MGUS). Patients from the Department of Clinical Therapeutics of the University of Athens were identified by review of the patient database of monoclonal gammopathies and includes serial MGUS/SMM data tracked by the Plasma Cell Dyscrasia department. Precursor patients from the UK were identified from the UCL Department of Hematology. Patients from the Czech Republic were identified from the Registry of Monoclonal Gammopathies (RMG) which includes disease diagnosis and risk and prognostic factors for precursor patients across several university hospitals. For more information on RMG, visit https://rmg.healthregistry.org/index.php.

Patients from all four sites were eligible for inclusion if diagnosed with non-IgM MGUS or SMM by the IMWG criteria. Patients diagnosed with overt MM at diagnosis were excluded from analysis, and patients treated with therapy during their precursor disease course were censored at treatment start dates. Patients were considered to have progressed from MGUS to MM or SMM to MM by the SLiM-CRAB criteria.[1] We conducted extensive quality control checks by performing Gaussian distributions on all biomarkers to identify outliers and verified the value of these outliers by electronic medical record review. The average time between date of original diagnosis and Visit 1 is 0 months for all cohorts (Training Cohort, Validation Cohort 1, Validation Cohort 2).

PANGEA Training Cohort

To develop a model that can predict progression to MM, we first trained Cox regressions on the PANGEA Training Cohort. We excluded patients initially diagnosed with MM, a subset of patients without progression status, and patients with IgM immunofixation. Additionally, we excluded data from lab visits that occurred after the date of progression to MM. For patients that were treated before progression to MM, we censored data from lab visits once they received

treatment. After applying these exclusions, the Training Cohort consisted of 1217 patients in total, with 172 progressing to MM.

Comparison to Existing Models

We evaluated the representativeness of our patient population by applying current risk stratification models of SMM and MGUS to the PANGEA Training and Validation Cohorts. The differences between Validation Cohort 1 and 2 allowed us to evaluate how the *PANGEA Model* performed in a precursor cohort with extensive longitudinal follow-up (Validation Cohort 1) and for precursor patients with limited data after onset (Validation Cohort 2). Specifically, we replicated the 2018 Mayo Criteria 20/2/20 findings[2] in SMM patients of the PANGEA Project and demonstrated that patients with zero 20/2/20 risk factors exhibit lower rates of progression than patients with one risk factor, followed by patients with two or more risk factors (**Appendix p. 12**). Additionally, we applied the IMWG criteria[3] for MGUS patients of the PANGEA Project and demonstrated that patients defined as high-risk progressed more rapidly to SMM than patients without high-risk features (**Appendix p. 13**).

The *PANGEA Model (BM* and *No BM)*

When evaluating models of progression risk on the Training Cohort, we evaluated a set of categorical and continuous variables. For categorical variables, we evaluated: history of hematological malignancy, race, sex, bisphosphonate treatment, immunofixation, and BMI. For continuous lab values, we evaluated: monoclonal protein, age, β2-microglobulin, creatinine, calcium, corrected calcium (total calcium + 0.8 * serum albumin), serum IgA, serum IgM, serum IgG, kappa free light chain (FLC), lambda FLC, FLC involve over uninvolved ratio, involved light chain, involved heavy chain, uninvolved light chain, LDH, albumin, and hemoglobin. We applied $\log_{10}$ transformations for the continuous variables of creatinine, calcium, corrected calcium, involved over uninvolved ratio, involved light chain, involved heavy chain, uninvolved light chain and LDH. We also examined categorical trend variables captured at each clinical visit and used current and previous measurements to determine if the biomarker presented similar measurements over time or if it was markedly increasing/decreasing. These categorical variables are defined through linear interpolation of the biomarkers' past measurements. After assessing the significance of the estimated biomarkers' trend (significance threshold set at 0·1), we

included a categorical trend variable for decreasing hemoglobin. Additionally, we tested production of the *PANGEA Model* (*BM* and *No BM*) with and without imputation in the Training Cohort and found minimal differences in C-statistics between these Models (<2% change in C-statistics); ultimately, we selected the Models that did not use imputation. For each predictor we also tested whether replacing the regression coefficient in the proportional hazard model with a more flexible function (i.e., f(x)) could capture potential non-linear relationships between predictor and progression to MM; an example of this is provided on page 19 of the Appendix. Importantly, none of the predictors used in the PANGEA Models improved prediction accuracy (increased C-statistic) in the Validation Cohorts when handled in this manner (**Appendix p. 11**).

To incorporate serial measurements and model trajectories of clinical biomarkers, we fit linear regressions to individual lab values at each visit date using data from a given visit and all prior visits. For each regression, we evaluated the significance of the t-test for the slope, and for regression results with p<0.1 and a positive slope, we set a trajectory indicator variable (trajij for variable i at timepoint j) to 1, and 0 otherwise. As this regression requires sufficient data to fit a linear regression, we set the trajectory variables to 0 for the first two timepoints for all variables for each patient. We then computed the trajectory variables at each time point for each lab value, and we added the trajectory variables as time-varying predictors to the Cox model.

Finally, we conducted bootstrapping and calibration analyses for each PANGEA Model. To assess uncertainty on the concordance (C-statistics) between predictions in Validation Cohorts 1 and 2, we used a straightforward bootstrap procedure consisting of two iterated steps: sampling with replacement the validation cohort (the size of the validation cohort and this resampled version are identical), and computations of C-statistics with splines for proportional hazards (see example in **Appendix p. 20**). Additionally, for Validation Cohorts 1 and 2, we evaluated the calibration of the PANGEA Models by computing the ratio between (i) number of predicted events (with event being progression from a precursor disease state to MM) between Visit 1 and Visit 2 and (ii) the number of actual events recorded (**Appendix p. 21**).

Data Handling

Few biomarker inputs were missing in the Training and Validation datasets, and we imputed some of them by following common practices such as conducted for the 20/2/20 model.[2] The

multivariable imputation by chained equations (MICE) package in R was used for all imputation processes with default settings. Specifically, if the BMPC % or the monoclonal protein were determined by the pathologist or SPEP, respectively, to be below the limit of detection ("Not Quantifiable"), then we set BMPC % to 1% (3% of all BMPC% in the Training Cohort) and M-spike to 0.01 g/dL (7% of all M-spikes in the Training Cohort) (the lower limits of these two tests). Similarly, we set BMPC % to 0% and M-spike to 0.00 g/dL if these variables were considered undetected in clinic. Overall, BMPC % was carried backwards for 90 days and forward for two years unless it was replaced by an antecedent or precedent measurement. For Validation Cohort 2, creatinine was missing at baseline for many patients, so we imputed this value using data on BMPC %, creatinine, age, monoclonal protein, and involved over uninvolved FLC ratio.

PANGEA Model (FISH)

For patients with both BMs and FISH panel results, all bone marrow biopsies after March 2020 had flow sorting performed with CD38, CD138, CD45, CD56, and CD319 probes. If ≥0.1% monotypic plasma cells were detected, FISH testing was performed as described in the PCPDS assay guidelines: https://www.mayocliniclabs.com/test-catalog/Overview/606079. Negative results were carried back and forward until replaced by a positive result, and positive results were carried forward. Positive results for primary events (+3/+7, +9/+15, trisomy 4, trisomy 12, trisomy 18, t(11;14), t(4;14), t(6;14), t(14;16), t(14;20), -13/13q deletion) were carried backward to baseline. Positive results for secondary events (-17/17p, +1q, and 8q24/MYC rearrangement) were not carried backward. All non-detected FISH findings were set as NAs and positive FISH results with total counts across the PANGEA Project < 20 were considered not powered and excluded from analysis. To create the *PANGEA Model (FISH)*, we added all available FISH findings as variables to the *PANGEA Model (BM)* through forward variable selection, and we assessed whether additional variables were significant in this multivariate model (Wald test $p<0.05$). FISH alterations that were not statistically significant included t(11;14), t(4;14), t(6;14), t(14;16), t(14;20) and hyperdiploidy as well as the combination of t(4;14), t(14;16), t(6;14), t(14;20) assessed together and t(4;14), t(14;16), 1q gain, -13/13q assessed together. Further, several FISH alterations (t(14;18), -6q, -11q22) were present in too few cases (N < 10) for analysis and were not included.

The PANGEA App

We created an interactive website to evaluate patient risk using the *PANGEA (BM)* or *PANGEA (No BM)* model. Users can enter the model variables (monoclonal protein, involved over uninvolved FLC ratio, creatinine, hemoglobin, and age). If BMbx data is available, users can enter this information and patient progression risk will be evaluated using the *PANGEA (BM)* model. Alternatively, if BMbx data is not available, users can enter all other variables, and patient progression risk will be evaluated using the *PANGEA (No BM)* model. If longitudinal measurements are available, users can enter variables at multiple time points. The *PANGEA Model* allows input of past and present patient measurements and outputs the probability of progression to MM at 1, 2, 5, 10, and 25 years regardless of whether the *PANGEA (BM)* or *PANGEA (No BM) Model* is used. If a variable is missing, a distribution of possible values for the missing variable based on the other variables is plotted, and users can enter different values for the missing variable to estimate the potential risk of progression.

References

1. Rajkumar SV, Dimopoulos MA, Palumbo A, et al. International Myeloma Working Group updated criteria for the diagnosis of multiple myeloma. *Lancet Oncol*, 2014; **15**:e538–48.

2. Lakshman A, Rajkumar SV, Buadi FK, et al. Risk stratification of smoldering multiple myeloma incorporating revised IMWG diagnostic criteria. *Blood Cancer J*, 2018; **8**:59.

3. Kyle RA, Durie BGM, Rajkumar SV, et al. Monoclonal gammopathy of undetermined significance (MGUS) and smoldering (asymptomatic) multiple myeloma: IMWG consensus perspectives risk factors for progression and guidelines for monitoring and management. *Leukemia*, 2010; **24**:1121–1127.

Supplementary Table 1. Distribution of laboratory measurements across 20/2/20 risk groups for the PANGEA Training Cohort.

			20/2/20 SMM RISK GROUPS			
		**MGUS**	**LOW**	**INTERMEDIATE**	**HIGH**	**Total**
**Number of Bone Marrow Biopsies**						n = 1217 (%)
Median (IQR)		1 (0 - 2)	1 (1 - 2)	2 (1 - 3)	2 (1 - 3)	
	0	307 (43)	6 (2)	4 (3)	5 (6)	
	1	214 (30)	167 (58)	55 (43)	25 (30)	
	2	95 (13)	56 (19)	28 (22)	30 (37)	
	3	55 (8)	36 (12)	18 (14)	12 (15)	
	4	22 (3)	18 (6)	12 (9)	8 (10)	
	5	11 (2)	6 (2)	7 (5)	-	
	6	7 (1)	-	1 (1)	1 (1)	
	7	3 (0)	1 (0)	2 (2)	1 (1)	
	8	3 (0)	-	-	-	
	9	1 (0)	-	1 (1)	-	
**Biopsy Conducted At Time of Diagnosis?**						
	Yes (%)	87 (20)	209 (89)	112 (91)	69 (87)	618 (51)
**Months Between Biopsies (averaged within patient)**						
Median (IQR)		2.1<br>(1.0 – 4.1)	1.3<br>(1.0 – 2.9)	1.0<br>(0.74 – 1.5)	1.0<br>(0.46 – 2.0)	
**Number of Laboratory Measurements**						
Median Creatinine (IQR)		5 (3 - 10)	6 (3 - 11)	5 (3 - 8)	4 (2 - 9)	
Median Hemoglobin (IQR)		5 (3 - 10)	6 (3 - 11)	5 (3 - 9)	4 (2 - 9)	
Median FLC Ratio (IQR)		5 (2 - 9)	6 (3 - 11)	5 (3 - 9)	4 (2 - 9)	
Median M-Spike (IQR)		4 (1 - 9)	6 (3 - 10)	4 (2 - 7)	3 (2 - 6)	
Median BMPC% (IQR)		1 (0 - 2)	1 (1 - 2)	2 (1 - 3)	2 (1 - 3)	

Supplementary Table 2. Patient FISH Characteristics in a PANGEA Subcohort.

	Total n = 6,445 (%)		DFCI n = 1,219 (19)	Greece n = 533 (8)	UK n = 109 (2)	Czech n = 4,584 (71)
**t(4;14)**						
No	899 (14)		874 (72)	-	25 (23)	-
Yes	117 (2)		62 (5)	9 (2)	3 (3)	43 (1)
Missing	*5,429 (84)*		*283 (23)*	*524 (98)*	*81 (74)*	*4,541 (99)*
**t(6;14)**						
No	946 (15)		921 (76)	-	25 (23)	-
Yes	14 (0)		11 (1)	-	-	3 (0)
Missing	*5,485 (85)*		*287 (24)*	*533 (100)*	*84 (77)*	*4,581 (100)*
**t(11;14)**						
No	851 (13)		822 (67)	4 (1)	25 (23)	-
Yes	210 (3)		124(10)		9 (8)	77 (2)
Missing	*5,384 (84)*		*273 (22)*	*529 (99)*	*75 (69)*	*4,507 (98)*
**t(14;16)**						
No	913 (14)		890 (73)	-	23 (21)	-
Yes	62 (1)		45 (4)	3 (1)	4 (4)	10 (0)
Missing	*5,470 (85)*		*284 (23)*	*530 (99)*	*82 (75)*	*4,574 (100)*
**t(14;20)**						
No	935 (15)		910 (75)	-	25 (23)	-
Yes	19 (0)		19 (2)	-	-	-
Missing	*5,491 (85)*		*290 (24)*	*533 (100)*	*84 (77)*	*4,584 (100)*
**t(14;18)**						
No	950 (15)		925 (76)	-	25 (23)	-
Yes	4 (0)		4 (0)	-	-	-
Missing	*5,491 (85)*		*290 (24)*	*533 (100)*	*84 (77)*	*4,584 (100)*
**-17/17p**						
No	926 (14)		899 (74)	2 (0)	25 (23)	-
Yes	91 (1)		43 (3)		-	48 (1)
Missing	*5,428 (84)*		*277 (23)*	*531 (100)*	*84 (77)*	*4,536 (99)*
**-6q**						
No	949 (15)		924 (76)	-	25 (23)	-
Yes	6 (0)		6 (0)	-	-	-
Missing	*5,490 (85)*		*289 (24)*	*533 (100)*	*84 (77)*	*4,584 (100)*
**- 11q22**						
No	952 (15)		927 (76)	-	25 (23)	-
Yes	4 (0)		3 (0)	-	1 (1)	-
Missing	*5,489 (85)*		*289 (24)*	*533 (100)*	*83 (76)*	*4,584 (100)*
**+1q**						
No	843 (13)		823 (68)	-	20 (18)	-
Yes	296 (5)		113 (9)	14 (3)	16 (15)	153 (3)
Missing	*5,306 (82)*		*283 (23)*	*519 (97)*	*73 (67)*	*4,431 (97)*
**8q24/ MYC rearrangement**						
No	937 (15)		912 (75)	-	25 (23)	-
Yes	20 (0)		19 (2)	-	1 (1)	-

Missing	*5,488 (85)*		*288 (24)*	*533 (100)*	*83 (76)*	*4,584 (100)*
**-13/13q**						
No	774 (12)		751 (62)	-	23 (21)	-
Yes	423 (7)		188 (15)	27 (5)	6 (6)	202 (4)
Missing	*5,248 (81)*		*280 (23)*	*506 (95)*	*80 (73)*	*4,382 (96)*
**+3/+7 hyperdiploid**						
No	866 (13)		841 (69)	-	25 (23)	-
Yes	94 (1)		93 (8)	-	1 (1)	-
Missing	*5,485 (85)*		*285 (23)*	*533 (100)*	*83 (76)*	*4,584 (100)*
**+9/+15 hyperdiploid**						
No	805 (12)		780 (64)	-	25 (23)	-
Yes	158 (2)		157 (13)	-	1 (1)	-
Missing	*5,482 (85)*		*282 (23)*	*533 (100)*	*83 (76)*	*4,584 (100)*
**+ 4**						
No	949 (15)		924 (76)	-	25 (23)	-
Yes	6 (0)		6 (0)	-	-	-
Missing	*5,490 (85)*		*289 (24)*	*533 (100)*	*84 (77)*	*4,584 (100)*
**+12**						
No	951 (15)		926 (76)	-	25 (23)	-
Yes	4 (0)		4 (0)	-	-	-
Missing	*5,490 (85)*		*289 (24)*	*533 (100)*	*84 (77)*	*4,584 (100)*
**+18**						
No	949 (15)		925 (76)	-	24 (22)	-
Yes	6 (0)		5 (0)	-	1 (1)	-
Missing	*5,490 (85)*		*289 (24)*	*533 (100)*	*84 (77)*	*4,584 (100)*

Supplementary Table 3. Performance of the *PANGEA Models* (*BM* and *No BM*) compared to the Rolling IMWG Models, as measured by C-statistic (95% confidence interval) using MGUS patients of Validation Cohort 2.

		**Baseline IMWG**	**Rolling IMWG**	**PANGEA Models**
Validation Cohort 2: Visit 1	**BM**	0.640 (0.500 – 0.807)	0.640 (0.518 – 0.718)	0.729 (0.643 - 0.941)
	**No BM**	0.667 (0.512 – 0.836)	0.670 (0.523 – 0.729)	0.879 (0.586 – 0.938)

Supplementary Table 4. Schoenfeld tests to evaluate the assumption of constant effects of the covariates on the risk of precursor disease progression to MM.

Predictor	Chi Square	DF	p-value
FLC Ratio (logged)	2.198	1	0.138
M-spike	1.456	1	0.227
Age	0.084	1	0.772
Creatinine (logged)	0.874	1	0.35
BMPC%	0.03	1	0.861
Hemoglobin Trajectory	0.175	1	0.676
Global:	5.752	6	0.452

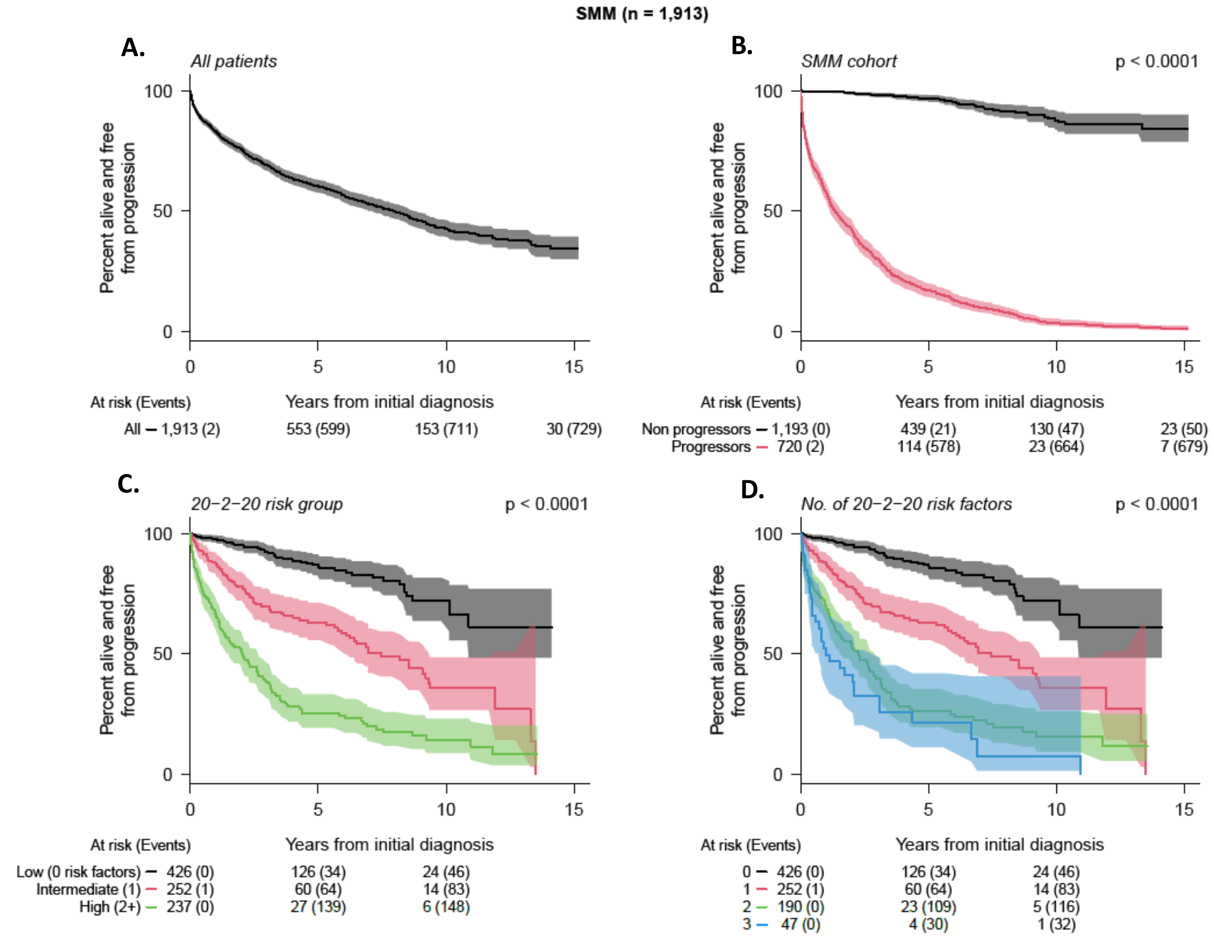

Supplementary Figure 1. PFS of all SMM patients in the PANGEA Project (Training Cohort, Validation Cohort 1, Validation Cohort 2). Kaplan-Meier curves indicating **A.** PFS of PANGEA SMM patients, **B.** PFS of patients who progressed from SMM to MM vs. non-progressors, **C.** PFS for SMM patients stratified by 20/2/20 risk group, and **D.** PFS for SMM patients stratified by 20/2/20 number of risk factors with separation of the high-risk group into patients with 2 risk factors vs. 3 risk factors.

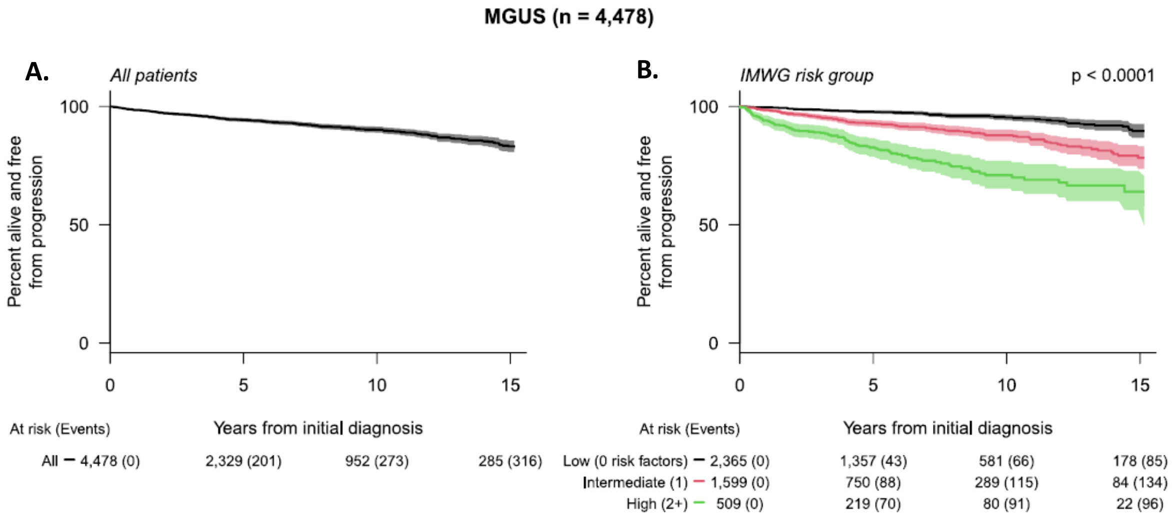

Supplementary Figure 2. PFS of all MGUS patients in the PANGEA Project (Training Cohort, Validation Cohort 1, Validation Cohort 2). **A.** Kaplan-Meier plot of PFS for all patients with MGUS diagnosis. **B.** PFS for all MGUS patients stratified by IMWG risk criteria.

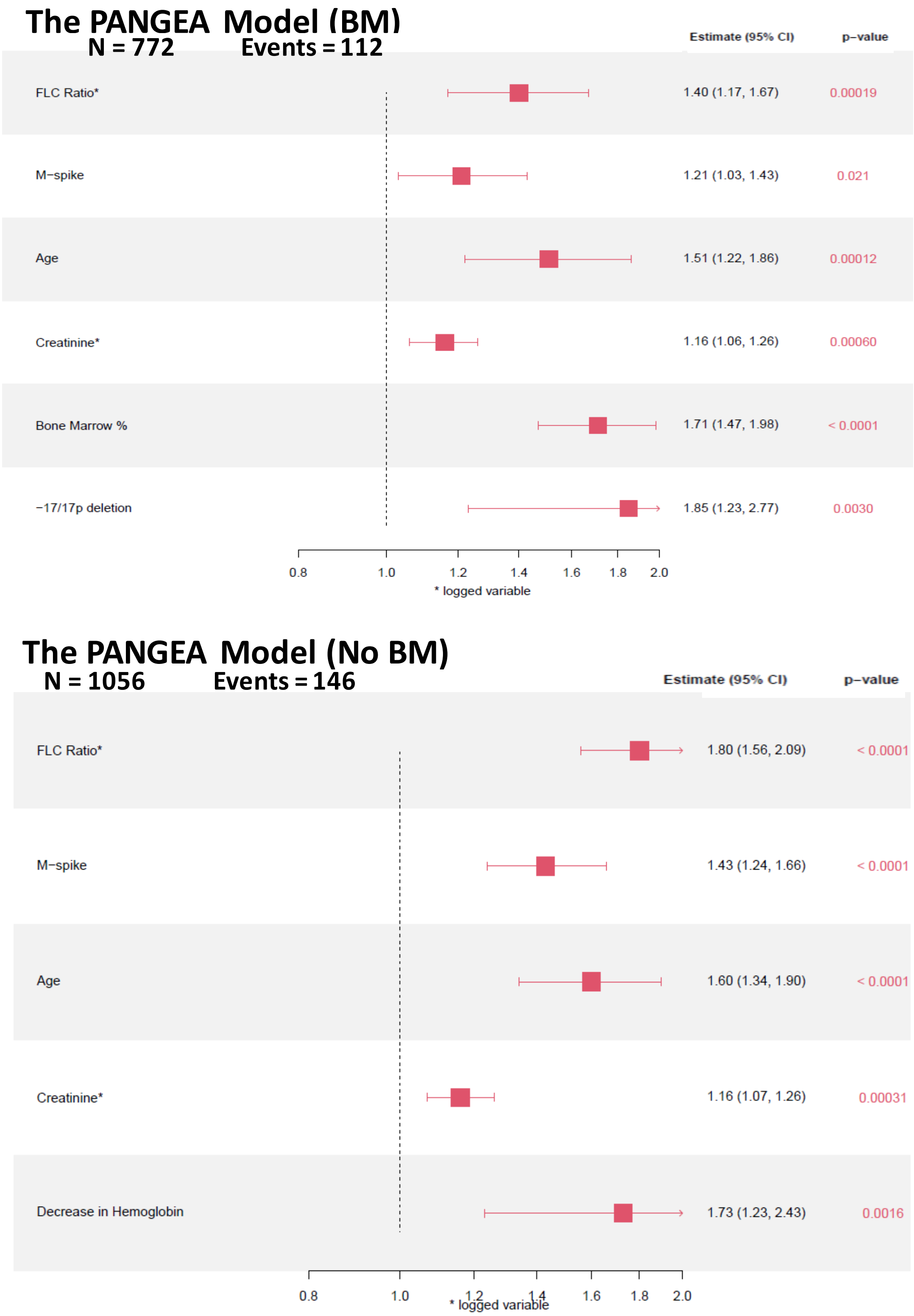

Supplementary Figure 3. Forest plots of clinical variables conferring precursor progression risk within the PANGEA Models. The *PANGEA (BM) Model* demonstrates risk predicted by FLC ratio, M-spike, age, creatinine, BMPC %, decrease in hemoglobin. The *PANGEA (No BM) Model* demonstrates risk predicted by FLC ratio, M-spike, age, creatinine, decrease in hemoglobin.

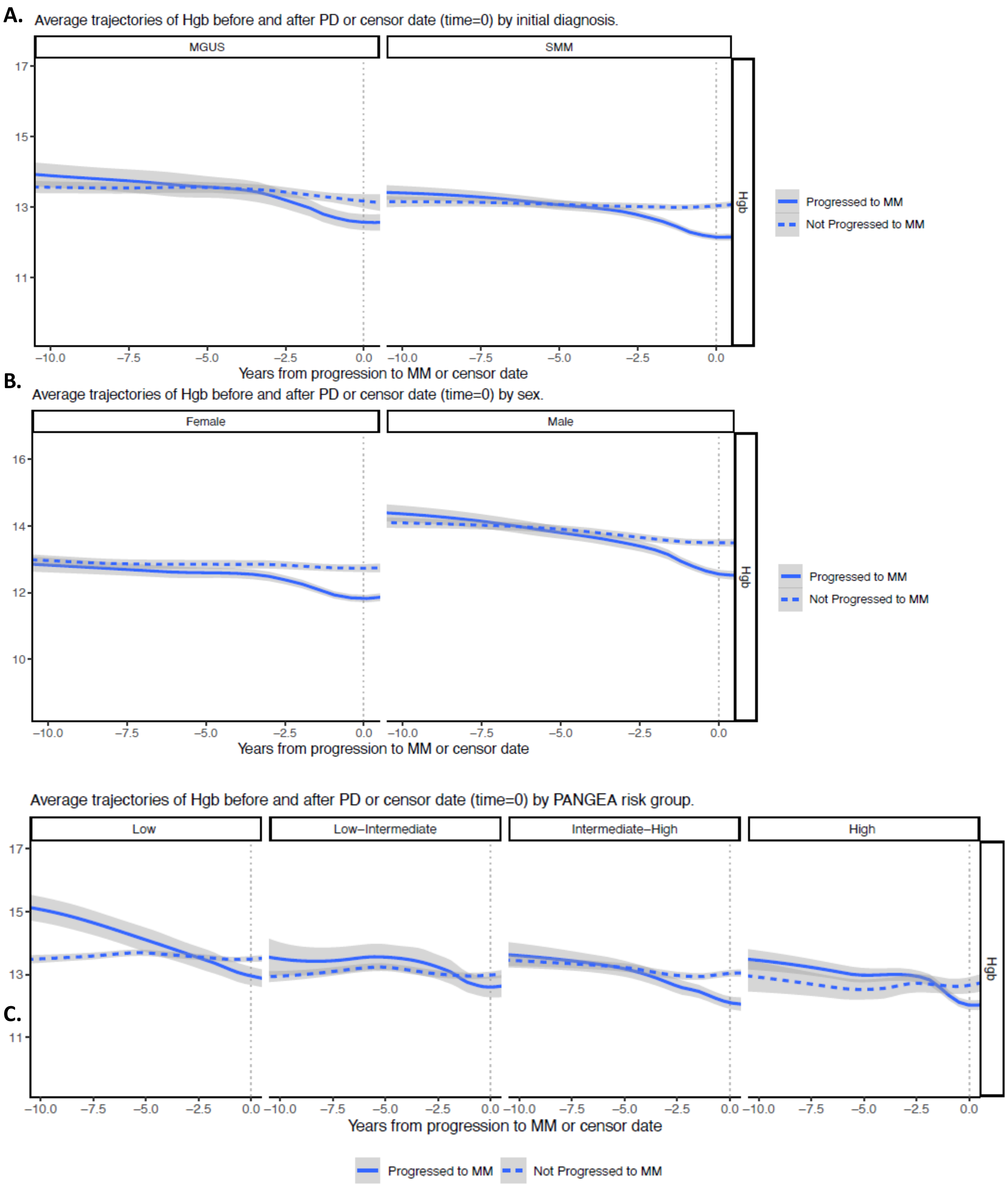

Supplementary Figure 4. Trended hemoglobin of the PANGEA Project for **A.** MGUS and SMM patients, **B.** male and female patients, and **C.** PANGEA risk groups.

A.

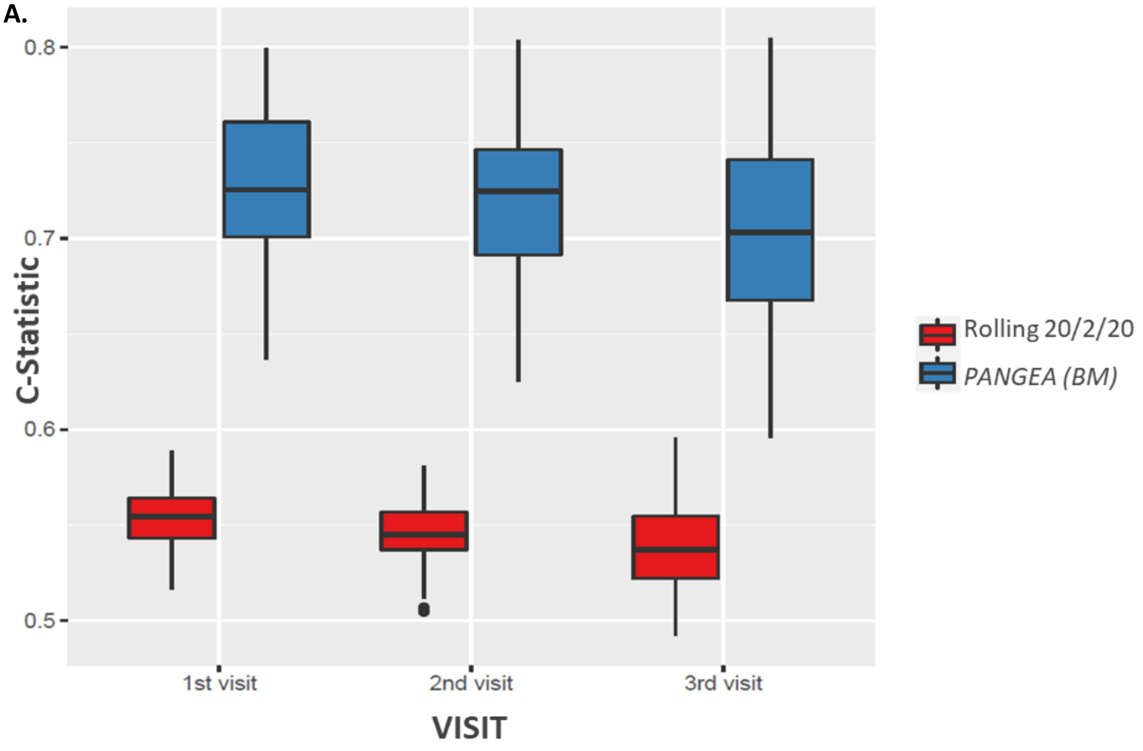

B.

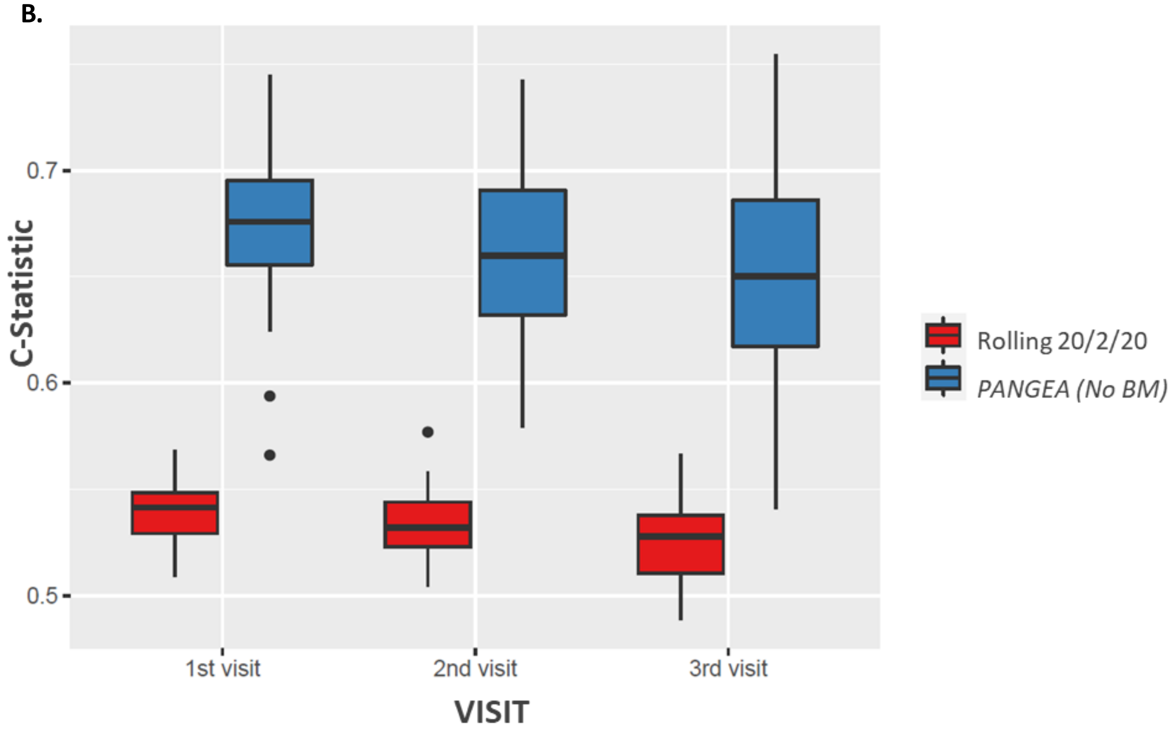

Supplementary Figure 5. Rolling 20/2/20 Model and **A.** *PANGEA (BM) Model*, **B.** *PANGEA (No BM) Model* evaluated using C-statistics and by performing bootstrap in the test data.

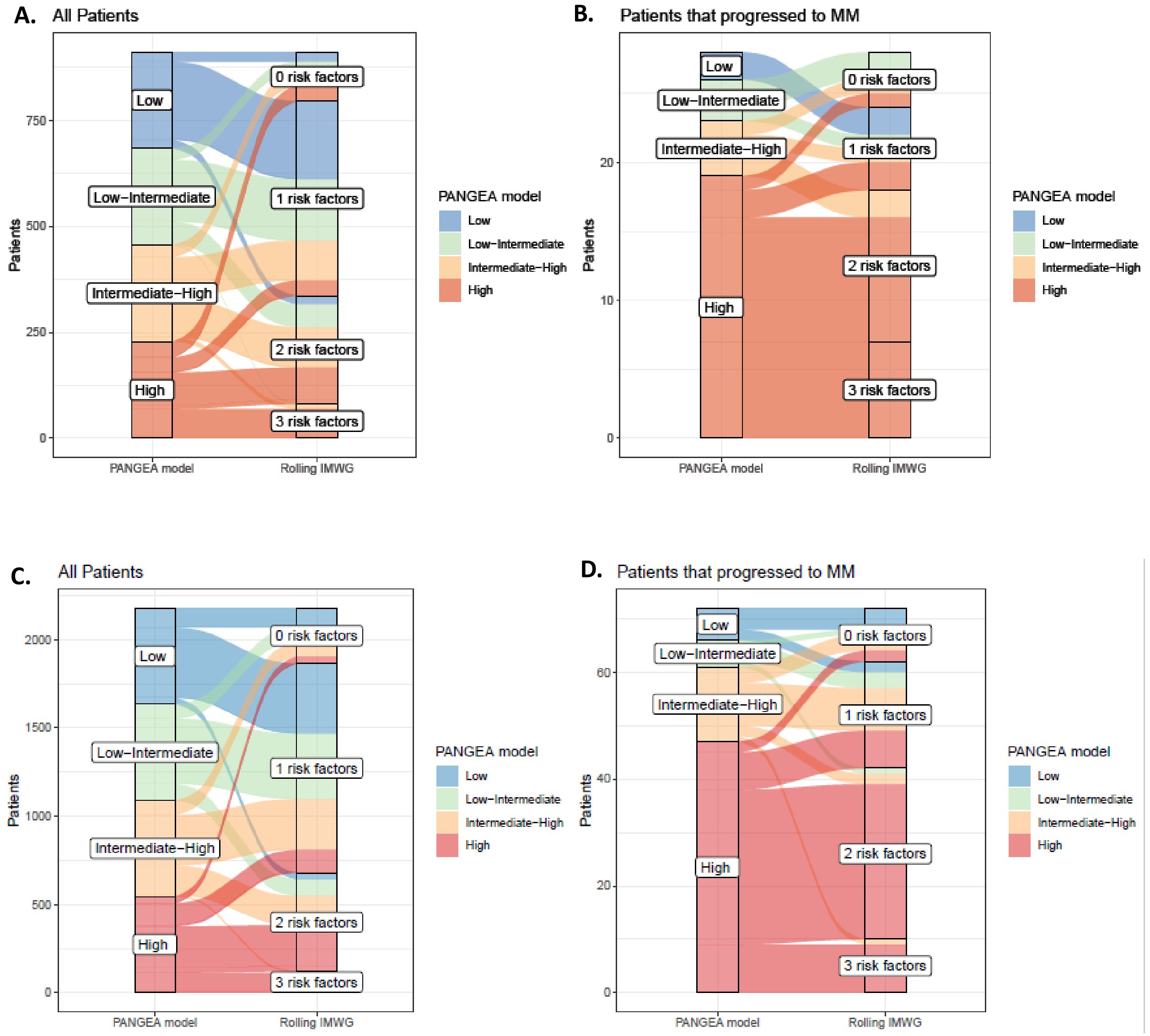

Supplementary Figure 6. Risk stratification of Validation Cohort 2 at First Visit for **A.** all MGUS patients and **B.** MGUS patients who progressed to MM, by the *PANGEA (BM) Model* compared to the *Rolling IMWG Model* and risk stratification of Validation Cohort 1 at First Visit for **C.** all MGUS patients and **D.** MGUS patients who progressed to MM, by the *PANGEA (No BM) Model* compared to the *IWMG Rolling Model.*

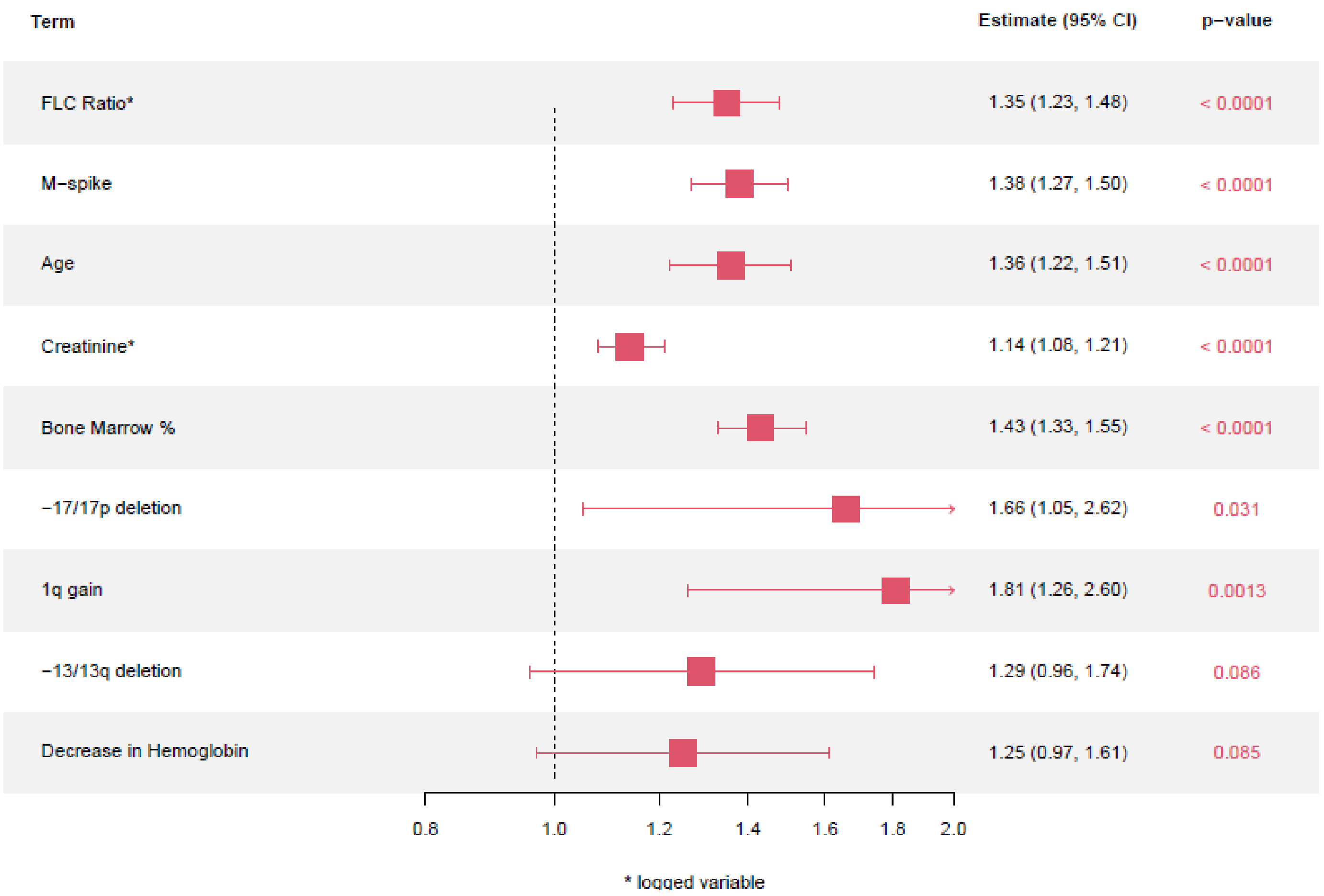

Supplementary Figure 7. Forest plot of risk factors associated with progression in the *PANGEA Model (FISH)* in a subcohort of patients who had FISH testing available.

.

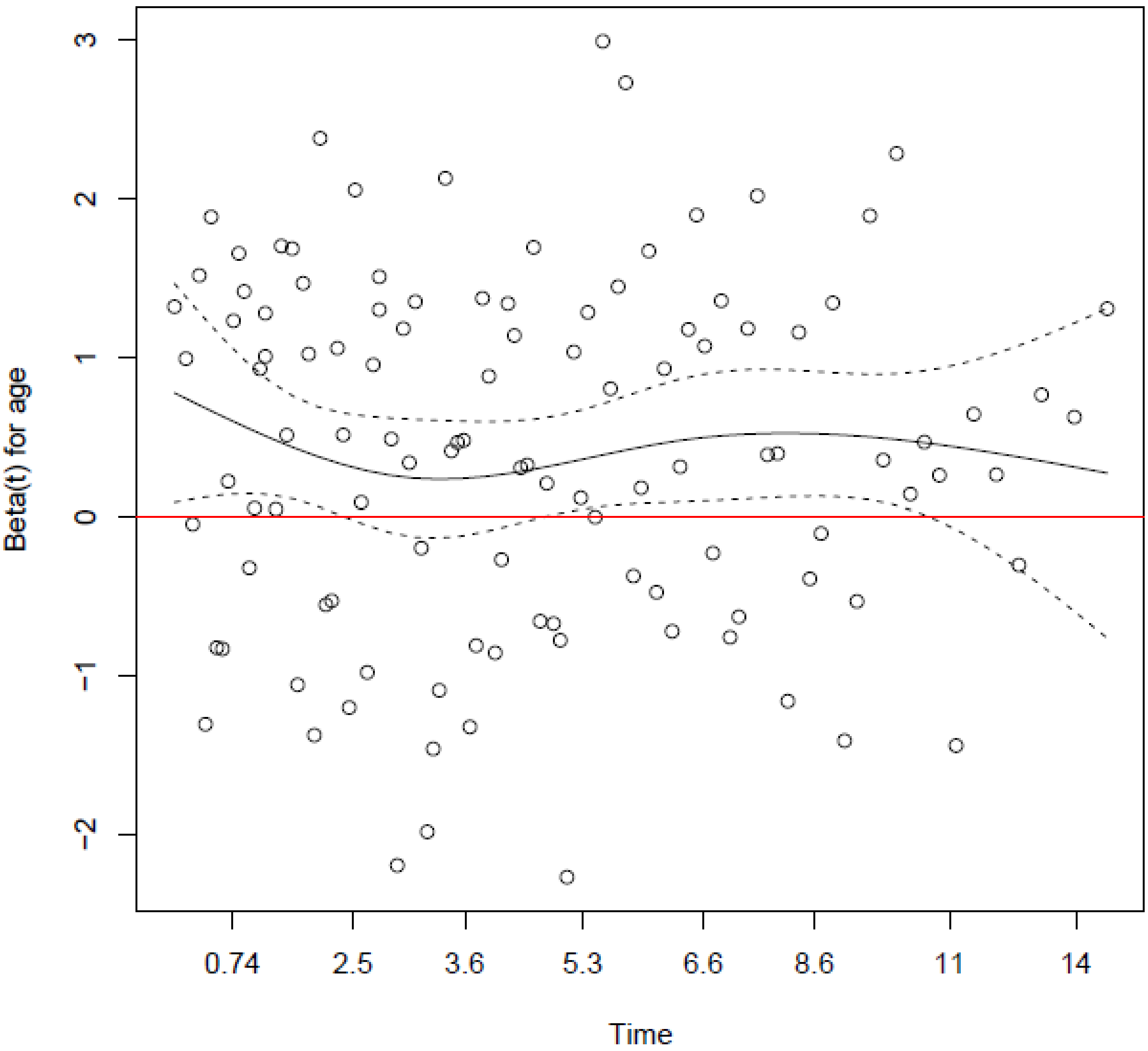

Supplementary Figure 8. Regression residual for the variable "age" of the *PANGEA Model (BM)* to investigate potential variations of the effects of age on the risk of progression over time.

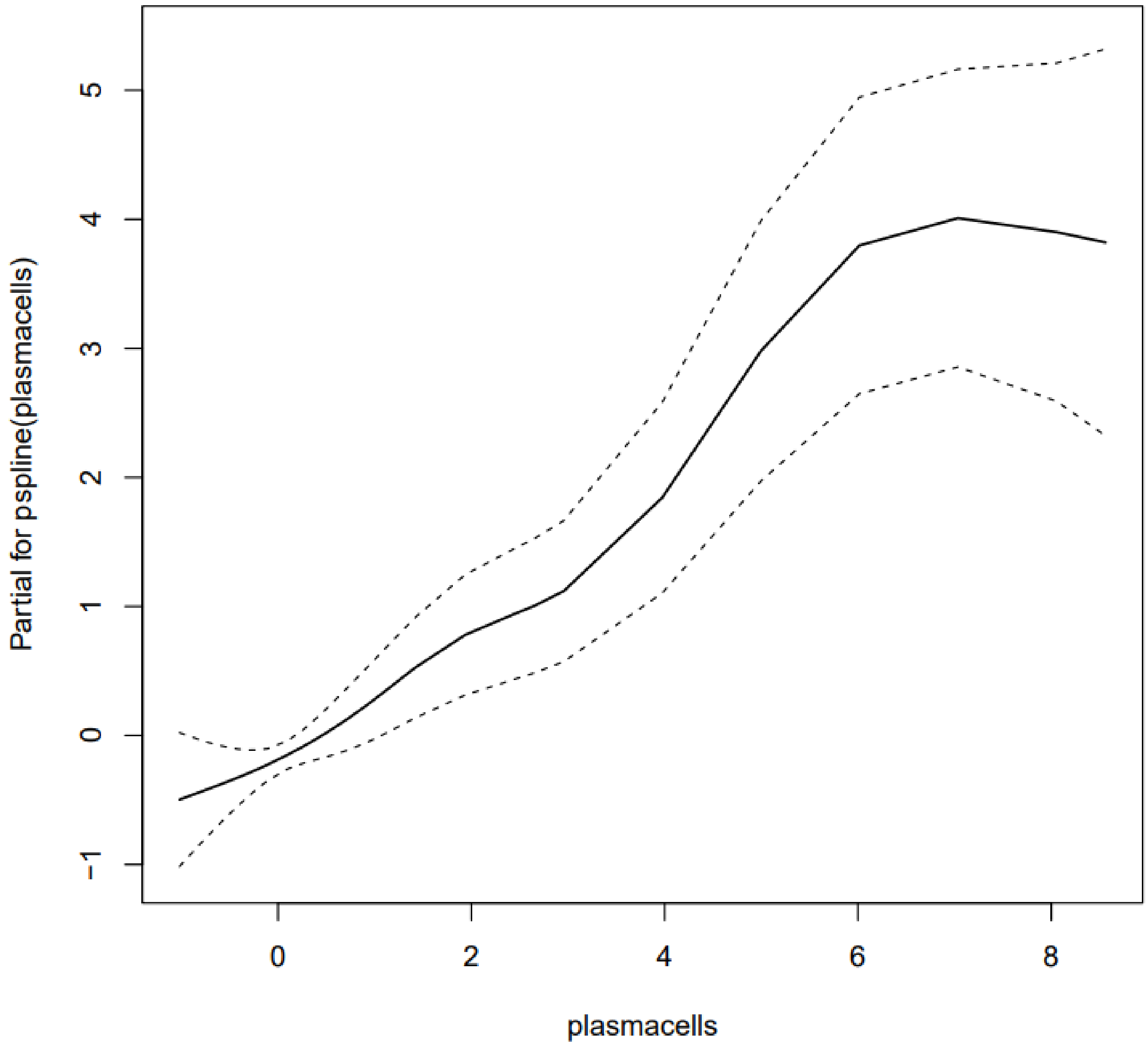

Supplementary Figure 9. The effect of the predictor "bone marrow plasma cell percentage" on the risk of progression. This graph illustrates the effects, estimated with a survival model that utilizes splines to capture the relation (log of relative risk) between the biomarker and the risk of progression. This is considered an extension f(x) of the linear relation of the PANGEA Model

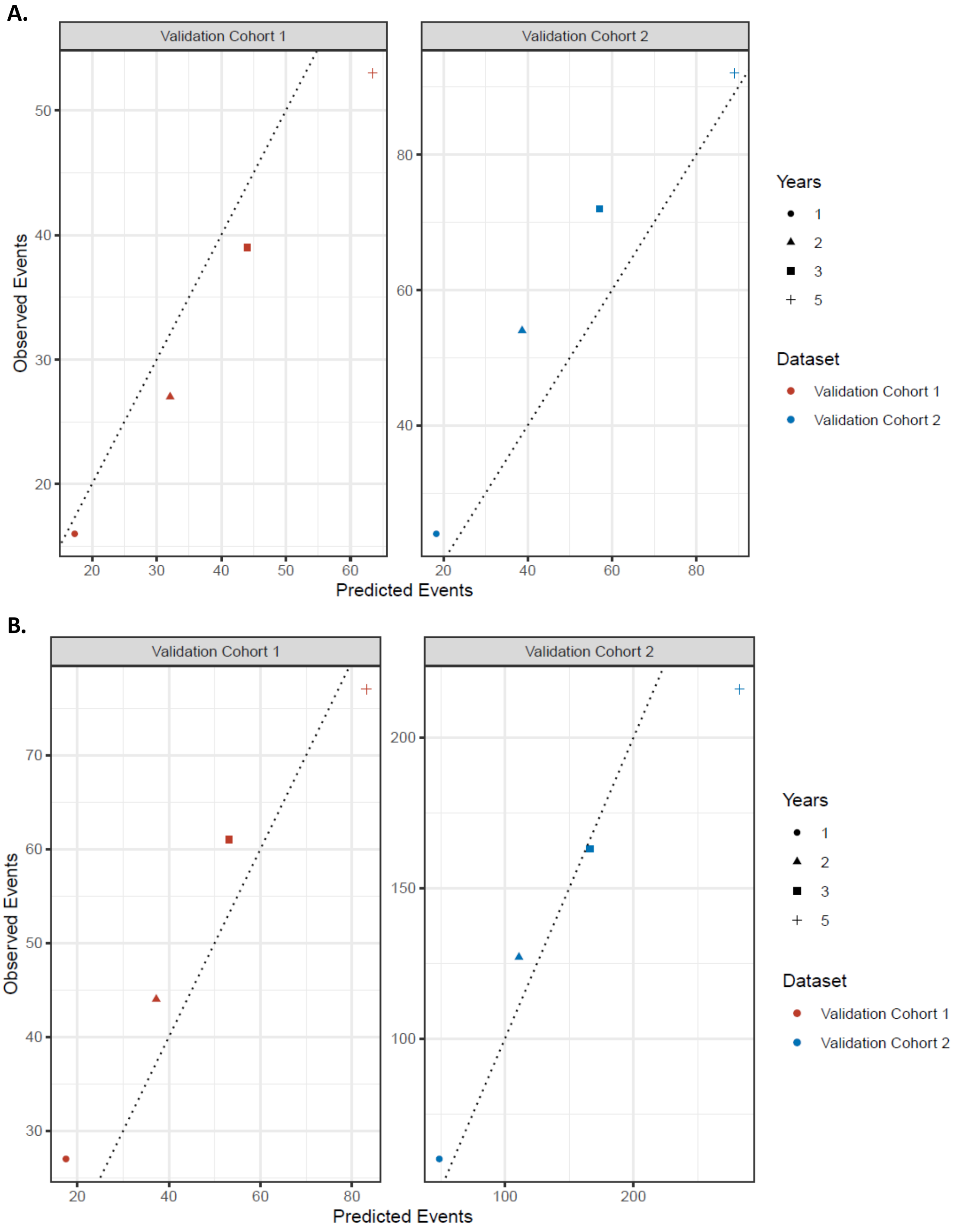

Supplementary Figure 10. PANGEA Model calibration in three independent datasets for the **A.** *PANGEA Model (No BM)* and **B.** *PANGEA Model (BM)*. Illustrated are the expected number of events and actual number of events at 1, 2, 3, and 5 years after Visit 1.
